# Evaluation of the Grafting Efficacy of Active Biomolecules of Phosphatidylcholine and Type I Collagen on Polyether Ether Ketone: In Vitro and In Vivo

**DOI:** 10.3390/polym13132081

**Published:** 2021-06-24

**Authors:** Jian-Chih Chen, Chih-Hua Chen, Kai-Chi Chang, Shih-Ming Liu, Chia-Ling Ko, Chi-Jen Shih, Ying-Sui Sun, Wen-Cheng Chen

**Affiliations:** 1 Department of Orthopedics, College of Medicine, Kaohsiung Medical University, Kaohsiung 807, Taiwan; d830191@yahoo.com.tw; 2 Department of Orthopedics, Kaohsiung Medical University Hospital, Kaohsiung 807, Taiwan; 3 Advanced Medical Devices and Composites Laboratory, Department of Fiber and Composite Materials, Feng Chia University, Taichung 407, Taiwan; nydiaa50925@gmail.com (C.-H.C.); ketty60221@gmail.com (K.-C.C.); 0203home@gmail.com (S.-M.L.); rayko1024.rb@gmail.com (C.-L.K.); 4 Department of Fragrance and Cosmetic Science, College of Pharmacy, Kaohsiung Medical University, Kaohsiung 807, Taiwan; cjshih@gap.kmu.edu.tw; 5 Drug Development and Value Creation Research Center, Kaohsiung Medical University, Kaohsiung 807, Taiwan; 6 Department of Medical Research, Kaohsiung Medical University Hospital, Kaohsiung 807, Taiwan; 7 School of Dental Technology, College of Oral Medicine, Taipei Medical University, Taipei 110, Taiwan; yingsuisun@tmu.edu.tw; 8 Dental Medical Devices and Materials Research Center, College of Dental Medicine, Kaohsiung Medical University, Kaohsiung 807, Taiwan

**Keywords:** polyether ether ketone, surface modification, osteogenic, type I collagen, phosphatidylcholine, in vivo

## Abstract

Biomolecule grafting on polyether ether ketone (PEEK) was used to improve cell affinity caused by surface inertness. This study demonstrated the sequence-polished (P) and sulfonated (SA) PEEK modification to make a 3D structure, active biomolecule graftings through PEEK silylation (SA/SI) and then processed with phosphatidylcholine (with silylation of SA/SI/PC; without SA/PC) and type I collagen (COL I, with silylation of SA/SI/C; without SA/C). Different modified PEEKs were implanted for 4, 8, and 12 weeks for histology. Sulfonated PEEK of SA showed the surface roughness was significantly increased; after the silylation of SA/SI, the hydrophilic nature was remarkably improved. The biomolecules were effectively grafted through silylation, and the cells showed improved attachment after 1 h. Furthermore, the SA/SI/PC group showed good in vitro mineralization. The new bone tissues were integrated into the 3D porous structures of SA/SI/PC and SA/SI/C in vivo making PEEK a potential alternative to metals in orthopedic implants.

## 1. Introduction

Polyether ether ketone (PEEK) is suitable for bone implant materials because of its good mechanical, chemical stability and thermal properties [1]. At present, PEEK has many clinical applications, such as spinal cages [2,3,4], phantom samples for skull prosthesis [5,6], and dental implants [6,7]. It can also be applied in upper jaw prostheses [8]. The biological inertness of PEEK prevents adverse reactions with tissues [9], but this may limit the applications of the implant because of its poor tissue affinity [10]. PEEK is still categorized as bio-inert; therefore, many studies have used different surface modification techniques to change its inherent inertness and address this shortcoming [11].

Many commonly used surface modification strategies to enhance bio-bonding forces are known, including sandblasting [12], plasma spraying [13], oxygen plasma [14], neutral atom beam [15], nitrogen plasma [16], and acid etching [17]. In addition to these physiochemical modifications, biological grafts can be used. The use of grafted active biomolecules generally aims to increase the bone–implant integration of PEEK, which is widely applied in orthopedic implants. Active biomolecules, such as type-I collagen (COL I) [18], peptides [19], and RGD [20], have been used in the medical field for surface modifications. In recent years, many approaches have been used to modify PEEK [21,22,23], such as the use polydopamine-coated PEEK substrates in grafting TiO_2_ onto the surfaces to increase its bioperformance and facilitate the integration of implants and tissues [22]. Another strategy used is depositing calcium phosphates into PEEK and further coating with antibiotics that have antibacterial and osteogenic activities [23].

In addition, studies on preparing PEEK with a 3D porous structure by sulfonation treatment are found. However, sulfonation treatment produces abundant sulfur-containing SO_3_H functional groups on the PEEK surface, which can release and display a negative influence on cell proliferation. Therefore, desulfonation by heating treatments or grafting biomolecules on PEEK are popularly used to improve the cell affinity. The results through hydrothermal treatment to get rid of the residual sulfur compounds indicate that 3D porous PEEK can induce the differentiation of M2 (alternatively activated macrophages) and promote tissue repair [24]. Torstrick et al. compared the osseointegration effects among porous PEEK, PEEK surface coating deposited with plasma-sprayed titanium, and PEEK with a smooth surface after being implanted into the tibia of rats [25]. Their results showed that all porous PEEK samples showed good osteogenic differentiation and osseointegration effects in vitro or performances after in vivo implantation. This study combines physical, chemical, and biomolecular grafting modifications by using sulfonation and silylation (silanization) to modify the surface structure and physiochemical properties of PEEK, and COL I and phosphatidylcholine are grafted to evaluate the surface modification effects of PEEK on the potential osseointegrated abilities to correlate in vitro conditions with the in vivo reality.

## 2. Materials and Methods

### 2.1. PEEK Control/Polishing Group Preparation

Medical-grade PEEK, which was purchased from Grand Ware Trading Co., Ltd. (Taipei, Taiwan), was processed based on our published research [26]. In standardizing the surface conditions of the PEEK sample, the samples were prepared at a flat disk with dimensions of 8 mm in diameter and 1 mm in thickness through a precision cutting machine (CL40, Top Tech Machines Co., Ltd., Taichung, Taiwan). Sliced PEEK surfaces were gradually polished with sandpaper to 2000 grit; further finished by 1, 0.3, and 0.05 μm alumina polishing powder slurry; and sequentially cleaned using water and 99.8% alcohol with ultrasonication for 15 min to remove any residues on the surface (the control P group).

### 2.2. Functionalized Porous Structure of Surface-Modified PEEK

The P group was immersed in a magnetically stirred concentrated sulfuric acid (98%, PanReac AppliChem, Chicago, IL, USA) and allowed to react for 30 min at room temperature (SA group). Afterward, the sulfonated SA samples were sequentially and ultrasonically cleaned with deionized water and acetone. After sulfonation, the samples were silanized with acetone containing 10% 3-mercaptopropyltrimethoxysilane (MPTMS; Sigma-Aldrich, St. Louis, MO, USA) for 30 min, further immersed in 1% glutaraldehyde with phosphate-buffered saline for another 30 min at 25 °C, rinsed in pure buffer saline, and then dried in a vacuum oven (the SA/SI group).

### 2.3. Bioactive Molecule Grafting

Solutions containing 5 wt% L-α-phosphatidylcholine (Sigma-Aldrich) or 0.025 mg/mL COL I (Type I collagen, Sigma-Aldrich) were prepared. Then, 60 μL of these solutions were dropped onto the surfaces of 8 mm φ test pieces. The PEEK samples were kept at 4 °C for 16 h for bioactive molecule grafting and dried. The groups with phosphatidylcholine and COL I were referred to as SA/SI/PC and SA/SI/C, respectively.

### 2.4. Surface Characterizations

The substrate was observed using an optical microscope (OM; Primotech, ZEISS, Jena, Germany), and the thickness of the sulfonated layer was measured using Matscope software (ZEISS). The measured substrate was randomly sampled into 250 pieces. The topographies of different groups were analyzed through scanning electron microscopy (SEM; S-3400, Hitachi, Tokyo, Japan).

The roughness test used a surface roughness tester (SJ-301, Mitutoyo Ltd., Kawasaki, Japan) to measure the centerline average roughness (Ra) of the samples after a series of PEEK modifications. Equation (1) for the measurements is as follows:

(1) $$\mathrm{Ra}=\frac{1}{L}\overset{L}{\underset{0}{\int }}│f(x)│\mathrm{dx}$$

The hydrophilic and hydrophobic properties were measured by a contact angle measuring instrument (CAMF100, Xuyang Nanotechnology Co., Ltd.; Creating Nano Technologies, Inc., Tainan, Taiwan) equipped with a charge-coupled device to observe the droplet shape for analysis.

The content of active biomolecules on the surface was tested using the Bicinchoninic Acid Kit (BAC, Sigma-Aldrich) as the test reagent, and the enzyme-linked immunosorbent assay ELISA macroplate reader (EZ Read 400, Biochrom, Cambridge, UK) was used to measure the absorbance at a wavelength of 570 nm. Furthermore, a standard curve was used to regress the content of active biomolecules. Put the sample into 48 wells and added 10 μL of each original standard solution (undiluted), then mixed with 200 μL of BCA reagent on the surface of the test sample and reacted in the oven at 60 °C for 15 min. Took 100 μL reaction solution and added it to 96 wells for absorbance to test the content of the biomolecules. Chemical analysis of the surface was performed by attenuated total reflection-Fourier transform infrared spectroscopy (ATR-FTIR; NICOLET 6700, Thermo, Agawam, MA, USA) to analyze the PEEK surface modifications.

### 2.5. Biocompatibility and Cell Morphologies

In accordance with ISO 10993-5:2009, the test cell used for cell viability was mouse fibroblast L929, and the culture medium used was minimum essential medium alpha (α-MEM; Gibco, Foster, CA, USA) containing 10% horse serum of fetal bovine serum (FBS; Gibco) and 1% antibiotics of penicillin/streptomycin (Gibco), which was placed in an incubator containing 5% CO_2_ at 37 °C.

Three control groups were identified in this testing, including the positive control (15% dimethyl sulfoxide, DMSO, Sigma-Aldrich), negative control (the extract obtained by immersing high-density polyethylene (HDPE) in the culture medium and placing it in an incubator for 24 h), and control (blank; culture medium only).

All samples were sterilized using an irradiation dose of 25 kGy gamma-ray and then tested. The extract of different PEEK specimens was prepared on the basis of the standard weight of the sterilized test specimen (1 g) immersed in the culture medium of 5 mL and placed in an incubator for 24 h. The cell viability quantitative test was performed to culture L929 cells in a 96-well plate at a density of 1 × 10^4^ cell/well overnight. Then, 100 μL/well of the extracts was added and cultured for another 24 h. The extract was aspirated, and afterward tetrazolium salt (XTT cell proliferation kit; Biological Industries, Beit Haemek, Israel) was added to 50 μL/well and 100 μL/well culture medium. The assay is based on the ability of metabolic active cells to reduce the tetrazolium salt XTT to orange colored compounds of formazan. The intensity of the dye is proportional to the number of metabolic active cells. After the plate was placed in an incubator for 4 h with XTT, an ELISA reader was used to test the absorbance at a wavelength of 492 nm. The absorbance was proportional to cell viability.

### 2.6. Relative Short-Term Cell Attachment

The bone marrow D1 cells from mesenchymal stem cells were cloned from BALB/c mice (American-type Culture Collection, Manassas, VA, USA). The cells were supplemented with 10% FBS/0.5% penicillin/streptomycin (Gibco). The cells were used before the eighth passage to prevent individual cell variation. The substrate disks of different PEEK surface condition groups were placed into 48-well plates at a contact cell density of 5 × 10^3^ cells/specimen and then shook for 1 h to allow D1 cell attachment. The cells were incubated for 1 h, 1 day, and 2 days. At different periods, the substrates were washed, fixed with 2.5% glutaraldehyde and paraformaldehyde, gold plated, and observed using SEM to determine the interval of attachment and proliferation for cell morphologies.

### 2.7. Relative Long-Term Cell Proliferation

D1 cells were cultured, and the alamarBlue^®^ reduction assay kit (BIO-RAD, Hercules, CA, USA) was used to analyze the proliferation rate of D1 cells. All samples were sterilized using 25 kGy gamma-ray and then cultured. The cells were cultured on different surface conditions of the PEEK sample at a concentration of 1 × 10^5^ cells/well. After 1 h, the cells were attached to the test piece, and 1 mL of medium was added. The culture time was 1, 4, 7, 10, and 14 days at different periods. After culturing, the PEEK substrates were washed twice with PBS, then mixed with cell culture medium with an AlamarBlue^®^ proliferation assay, and incubated at 37 °C for 4 h. One hundred microliters of culture medium was placed on a 96-well plate and measured with an ELISA reader. The cell numbers were determined from a plot of absorbance (OD values) versus the respective D1 cells and spectrophotometrically measured at 570 and 600 nm. Each experiment was performed five times (*n* = 5).

### 2.8. Alkaline Phosphatase (ALP) Semi-Quantification and Staining

The cell mineralization of ALP production on the surfaces of different PEEK surface groups was determined using the *p*-nitrophenyl phosphate (*p*-NPP) kit (Sigma-Aldrich, Darmstadt, Germany) with Tris-buffered saline, and ALP staining was performed using the SIGMA*FAST*™ BCIP^®^/NBT kit (Sigma-Aldrich, Darmstadt, German) in accordance with the manufacturer’s instructions. Testing was performed simultaneously with the same intervals as in the cell proliferation tests. ALP activity was determined through absorbance measurements using an ELISA reader at 405 nm.

### 2.9. In Vivo Studies

The animal study was reviewed and approved by the Institutional Animal Care and Use Committee (IACUC) of Kaohsiung Medical University (IACUC 104091, 1 June 2016). The experimental groups were P, SA, SA/SI/PC, and SA/SI/C, and the implantation time was 4, 8, and 12 weeks. The surgical procedure was the same as that of the previous study [27,28]. The surface-modified PEEK cylinder with a diameter of 4 mm and height of 5 mm was implanted into the created bone defect using a drill with a diameter of 4 mm, and depth stops of 5 mm were made in the distal medial malleolus (condyle) of the rabbit femur. The experimental rabbits were sacrificed at 4, 8, and 12 weeks after implantation. After the femurs with implants were fixed and dehydrated, these non-decalcified tissues were then cold embedded in epoxy resin. The embedded non-decalcified test specimen was cut into thin slices by a slow cutting machine. The thickness of the test piece was gradually reduced to 500 µm by grinding, and then, the test specimen was pasted to the carrier. The glass slide was polished two times until the thickness of the test specimen was 200 µm and finally polished with alumina slurry of different particle sizes to complete the non-decalcified tissue specimen. The completed test specimen was stained with hematoxylin and eosin (H&E), and histological observation was performed with an OM and image analysis software.

### 2.10. Statistical Analysis

Statistical analysis was performed through IBM SPSS Statistics 20 (IBM Corporation, Armonk, NY, USA), and one-way analysis of variance (ANOVA) was used to determine statistically significant differences with *p* < 0.05. Comparison procedure using Tukey’s Honestly Significant Difference test was performed to analyze significant differences among the groups [29].

## 3. Results and Discussion

### 3.1. Surface-Modified Characterizations

#### 3.1.1. Grafting Concepts, Substrate Structures, and Surface Roughness

**Figure 1:**
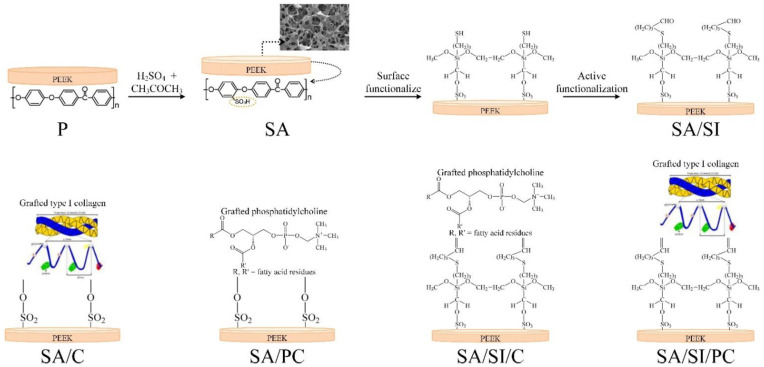
Schematic diagram of biopolymer grafting with PEEK surface modification.

The schematic diagram of the proposed reactions by surface chemical modification is shown in Figure 1. The polished PEEK with controlled surface topography (6.8 ± 0.4 nm with mean ± standard deviation) was first sulfonated so that the surface of the PEEK would obtain sulfonate groups and a microporous structure. Then, the sulfonated PEEK was silanized so that the surface of the specimen would obtain aldehyde groups to facilitate the grafting of biomolecules. Next, the sulfonated surface-treated PEEK with and without silylation was further grafted with COL I and phosphatidylcholine (Figure 1). The proposed progressive bonding procedure was hydrolysis of silane in an alcohol-containing aqueous solution to produce molecular groups with hydroxyl (R–CH_2_–OH). This molecular group was combined with the sulfonic acid (–SO_3_H) produced by sulfonation to obtain the thiol derivative bonding of R–SH on the PEEK surface. This molecule was aldehyde fixed to form an aldehyde group (–CHO) [30], allowing the amine or carboxylic acid ester groups of active biomolecules to bond with the aldehyde and grafting the imine or carbonyl bonding between the active biomolecules and PEEK substrate surface (Figure 1).

**Figure 2:**
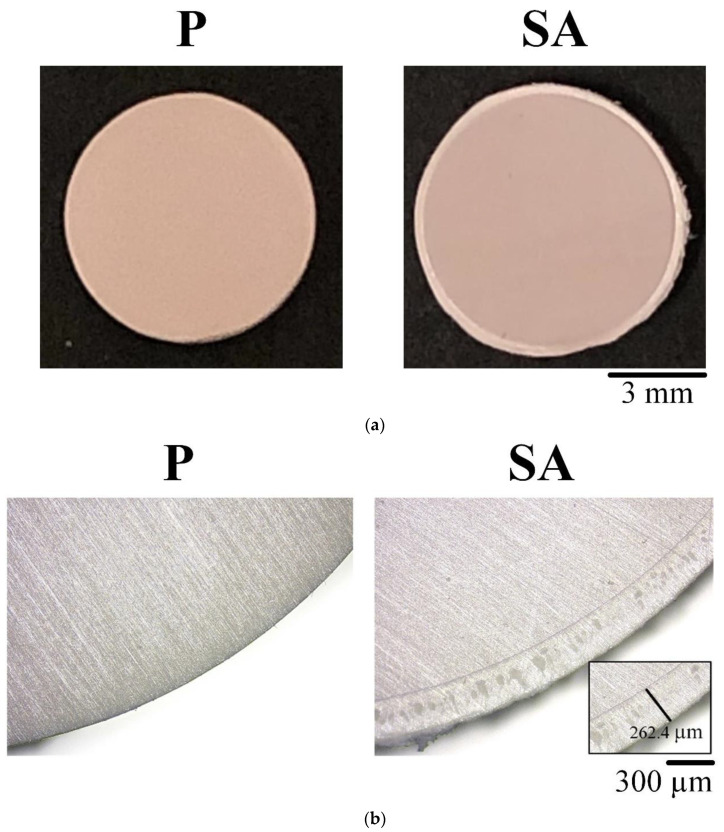
Cross-sectional observations of unmodified PEEK of the P group and sulfonated PEEK of the SA group: (**a**) photos and (**b**) OM images.

**Figure 3:**
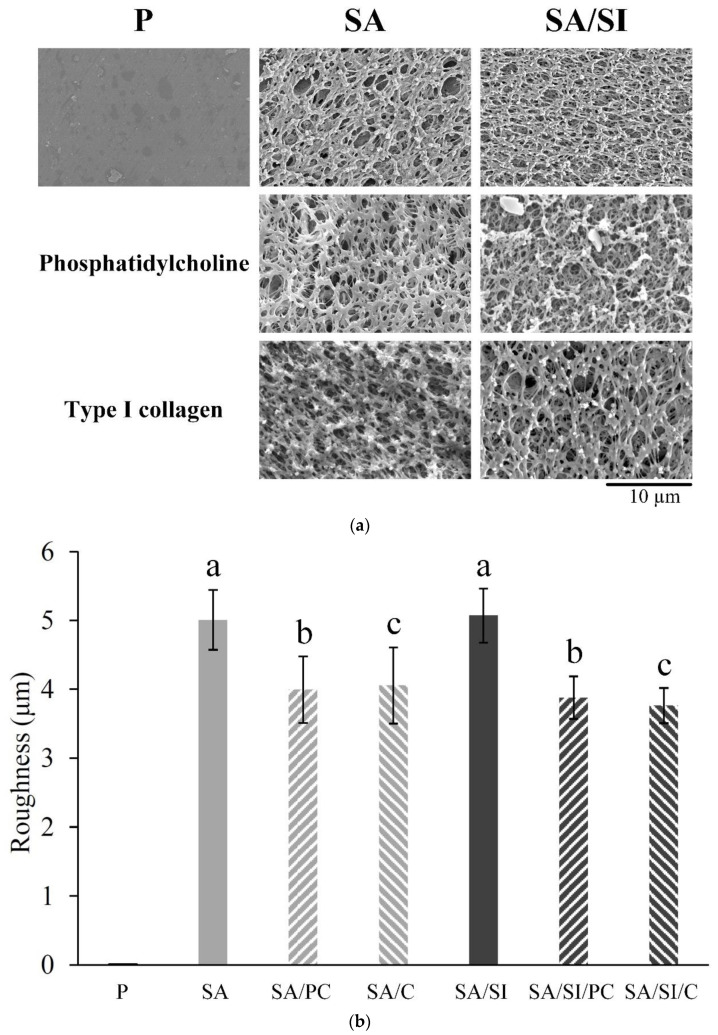
Different surface-modified PEEK measurements of (**a**) microscopic morphological observation and (**b**) roughness tests (*n* = 6). Character symbols a, b, and c indicate that each testing group after one-way ANOVA is not significantly different (*p* > 0.05).

The sample dimension used in this experiment after PEEK sulfonation would expand, which increased in size and varied slightly with an outer diameter of approximately 8.28 ± 0.03 mm from 7.99 ± 0.02 mm (Figure 2a). Under OM observation, a sulfonated layer appeared around the test specimen (SA), and the thickness of the sulfonated layer was 269.35 ± 42.34 μm (*n* = 250; Figure 2b). The PEEK surface had evenly distributed and inter-connective micropores after sulfonation under SEM observation (Figure 3a), and the surface roughness was also significantly increased (Figure 3b). After silanization of the sulfonated PEEK (SA/SI), the surface pore structure and surface roughness had no evident changes. After further grafting of active biomolecules (SA/SI/PC, SA/SI/C), no evident difference was observed in pore types (*p* > 0.05), although the roughness decreased slightly.

#### 3.1.2. Surface Wettability and Bioactive Molecule Grafting

**Figure 4:**
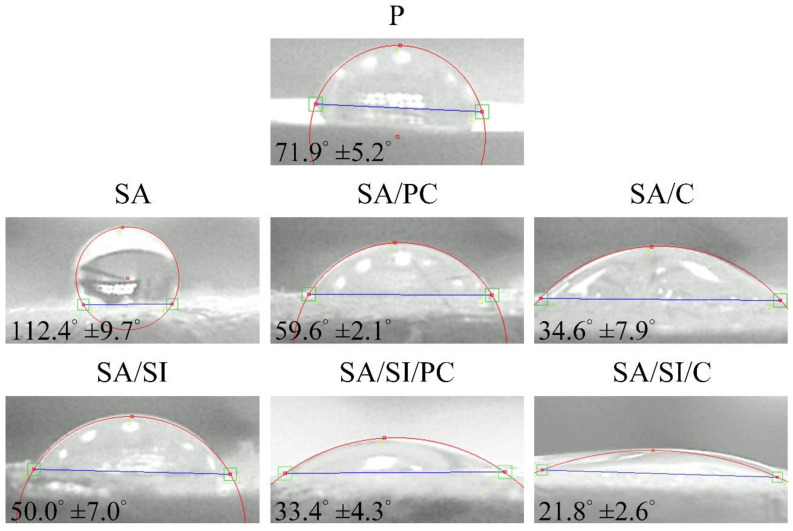
Wetting angles of different surface-modified PEEK hydrophilicity tests (*n* = 5).

The water droplet contact angle of surface-modified PEEK (Figure 4) showed that the contact angle of p was 71.9°. After sulfonation (SA), the porous structure and roughening of the surface resulted in an increase in the contact angle showing a hydrophobic surface. After silylation (SA/SI), the contact angle was decreased to 50° because of hydroformylation, which caused the formation of aldehydes on the surface; thus, the substrates changed to hydrophilic. In the molecular structure of phosphatidylcholine, one end was a hydrophobic fatty acid chain, and the other was a hydrophilic choline end. Given the hydrophilic choline end, water wettability increased, and among the groups, SA/SI/PC provided bonding to graft with more phosphatidylcholine compared with SA/PC without silylation. Therefore, the SA/SI/PC group was more hydrophilic than SA/PC. COL I was grafted with silylation (SA/SI/C), which had the same trend as SA/C, and COL I could effectively improve the hydrophilic nature of the substance because its structure contained many amino acids, such as RGD sequence [31].

**Figure 5:**
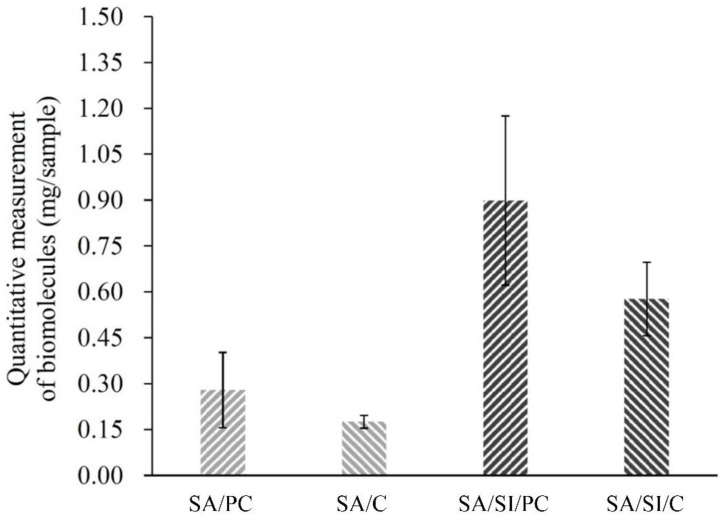
Bioactive molecule grafting weights of different surface-modified PEEK surfaces (*n* = 6).

In the graft weights for phosphatidylcholine and COL I (Figure 5), the SA/SI surface could be efficiently grafted with active biomolecules, and the weight content of the grafted phosphatidylcholine was higher than that of grafted COL I. Considering that functional aldehydes were produced after silylation, which could form amide bonding sides with active mbioolecules, more active biomolecules were grafted than groups without silylation. The groups after sulfonation/silylation obtained grafts three times higher than the groups that only underwent sulfonation, indicating that silylation-functionalized PEEK could effectively graft the active biomolecules.

**Figure 6:**
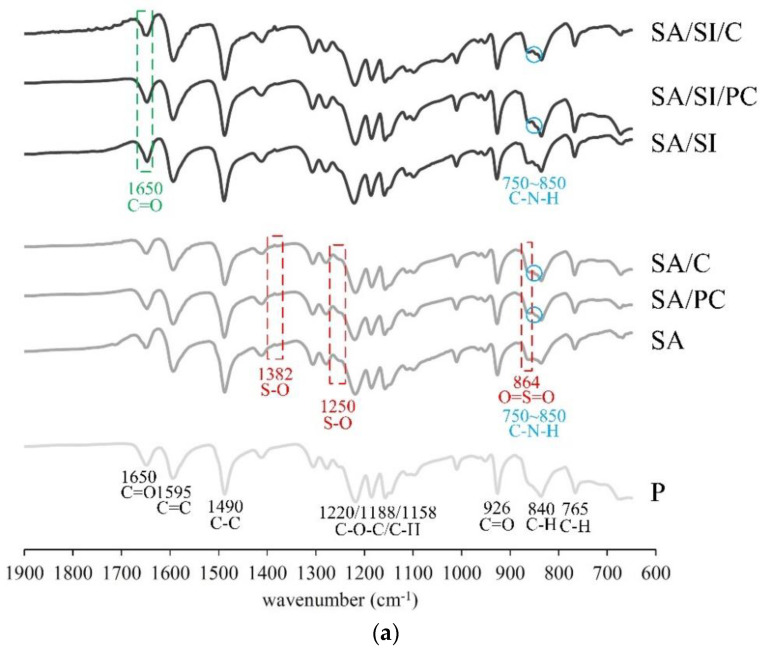

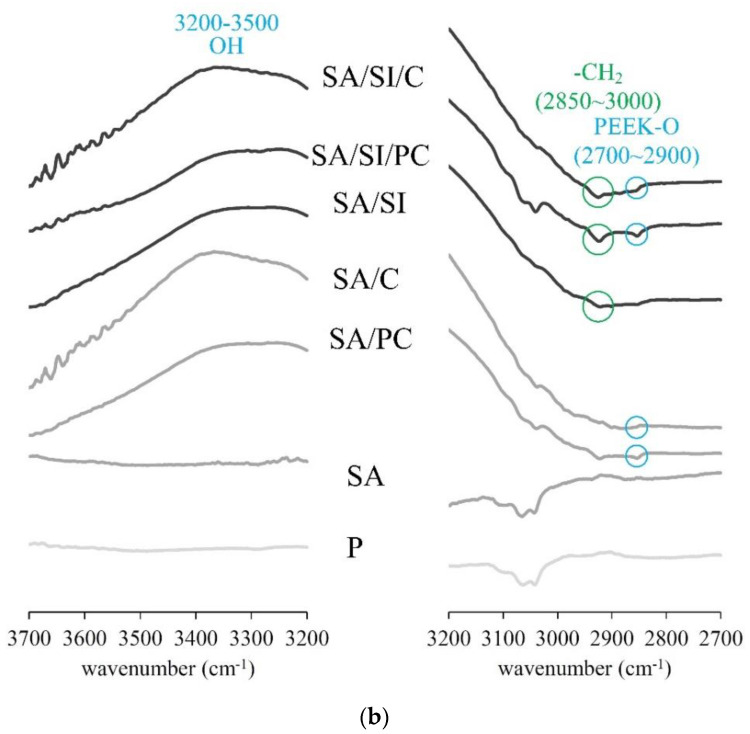
FTIR spectrum of different surface-modified PEEK under (**a**) approximately fingerprint absorption zone and (**b**) specific functional group absorption zone.

The surface-modified PEEK was analyzed by ATR-FTIR (Figure 6), and the spectrum showed CH stretching vibrations at 765 and 840 cm^−1^, C=C stretching at 1595 cm^−1^, CH bending vibrations at 1158 and 1188 cm^−1^, and C–C vibration at 1490 cm^−1^. Such findings were all aromatic characteristic absorptions of the ring vibration [32,33]. The stretching of 1220 cm^−1^ C–O–C was the vibration peak of ether connected between two aromatic rings; the C=O vibration peak of 926 cm^−1^ and the stretching of 1650 cm^−1^ were the ketone groups. The above-mentioned absorption peaks were typical vibration peaks of PEEK [34]. In the group after sulfonation, the vibration peak at 864 cm^−1^ was the absorption of sulfonate (SO_3_H) in O=S=O symmetric stretching [35]; absorptions at 1250 and 1382 cm^−1^ were the SO asymmetric stretching in sulfonate vibrations [35,36]. This result also showed that PEEK reacted with sulfuric acid after sulfonation to form sulfonic acids. After silylation, the characteristic C=O peak appeared at 1650 cm^−1^, and the stretching vibrations of amide I should appear at 1647 cm^−1^ for bioactive molecule grafts. However, these two absorptions were the same as the vibrations of the ketone in PEEK, which caused them to overlap, and verifying became difficult. The –CH stretching vibration of 2850–3000 cm^−1^ was primarily absorbed by the propyl group in MPTMS [37]. The OH functional groups at 3200–3500 cm^−1^ were observed, indicating that the aldehyde was generated during hydroformylation in the silylation [35]. The characteristic band of the grafted bioactive molecule PEEK-O was observed at 2700–2900 cm^−1^.

### 3.2. Biocompatibility and Measurements In Vitro

#### 3.2.1. Cell Viability of Fibroblast L929 Cells

**Figure 7:**
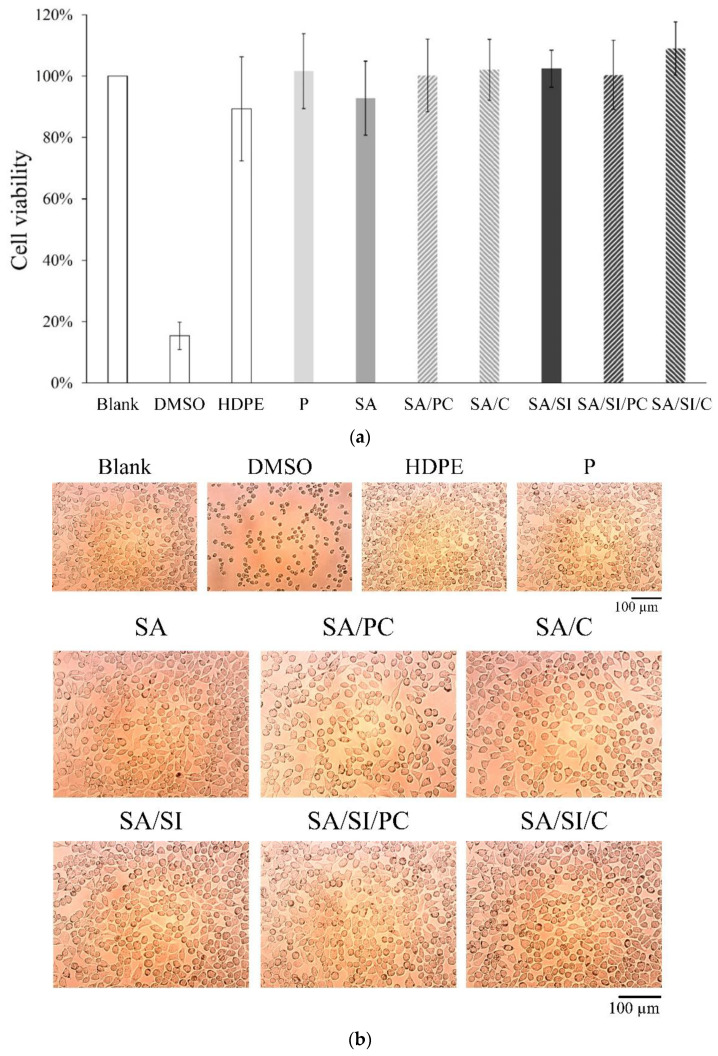
Surface-modified PEEK cytotoxicity test: (**a**) cell viability indicates the proportion of live and healthy cells within a population under cell viability assay analysis (*n* = 6) and (**b**) optical images of cell morphological observation.

The specification standard in the ISO-10993-5, which regulated the cytotoxicity testing, indicated that the cell viability was higher than 70%, which implied that the material was not cytotoxic. The results among the three control and seven experimental groups (Figure 7a) showed only the cytotoxic positive control group DMSO, whose cell survival rate was only 15%.

This result also showed that PEEK was not toxic to cells even after different surface modifications and grafting of active biomolecules. The cells in the 15% DMSO positive control group aggregated and showed a spherical shape, indicating cell death (Figure 7b). In the negative control group of HDPE the cells were healthy and spindle shaped similar to the control group, indicating that sterilization was effective. Optical microscopy analysis mainly depends on whether the cell phenotype is affected (Figure 7b). Semi-quantitative cell number analysis is more accurate for cell proliferation (Figure 7a). The experimental groups showed that apart from the cell survival rate, which have met the standard, the cells had no adverse effects such as degeneration compared with those of the control group.

#### 3.2.2. Cell Attachment of Bone Progenitor D1 Cells

**Figure 8:**
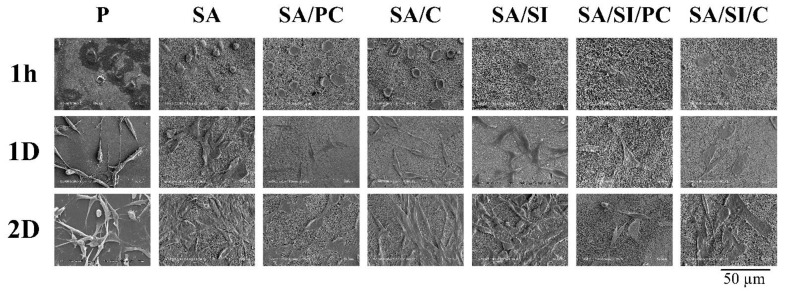
Microscopic images of progenitor D1 cell attachments on surface-modified PEEK for the culture periods of 1 h, 1 day, and 2 days.

Unmodified PEEK had some D1 cells attached on the substrate surface within 1 h, yet more cells showed incompletely attached spherical morphology (Figure 8). By contrast, the sulfonated (SA) group had a more hydrophobic surface, causing the unattached spherical shape of the cells. The cell morphologies of the groups (SA/PC and SA/C) grafted with active biomolecules after sulfonation without silylation were in the form of preliminary attachment. In the groups modified by sulfonation with further silylation (SA/SI, SA/SI/PC, and SA/SI/C), the cells displayed flat membrane topography, and some cells formed pseudopodia because these groups had both hydrophilic and rough surface properties [38,39,40] so that the cells could complete attachment in a short time.

After 1 day of culture, the cells of all groups were attached to the surface of the PEEK, but the cells of the silylated groups (SA/SI, SA/SI/PC, and SA/SI/C) were flat, and the area of cell attachments was wider than that of other PEEKs. After 2 days of cell culture, the cells on the surface of unmodified PEEK still showed a longitudinal thin shape, whereas the cells of other groups were proper spindle flat with good attachment. This result showed that after sulfonation, PEEK could promote cell attachment because of its surface roughness and PEEK after sulfonation modified with silylation. It could not only promote cell attachment but also attract cell attachment in the early stage.

#### 3.2.3. Cell Proliferation of Bone Progenitor D1 Cells

**Figure 9:**
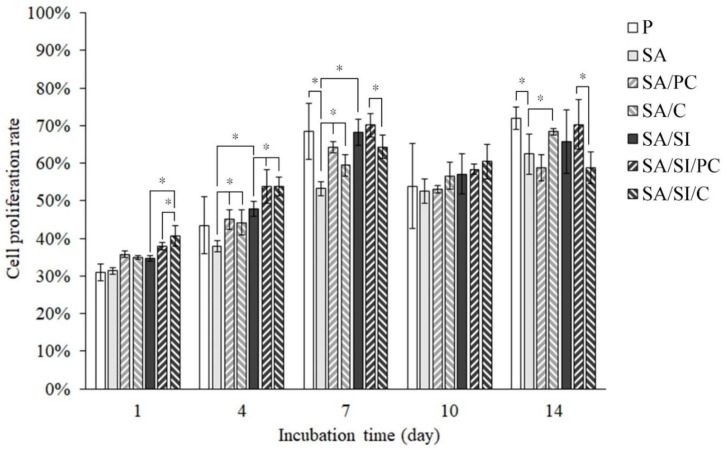
Cell metabolic proliferation assay analysis (AlamarBlue^®^ kit) of the D1 cell proliferation test on surface-modified PEEK for 1, 4, 7, 10, and 14 days (*n* = 6). Character symbols * indicate that each testing group after one-way ANOVA is significantly different (*p* < 0.05).

**Figure 10:**
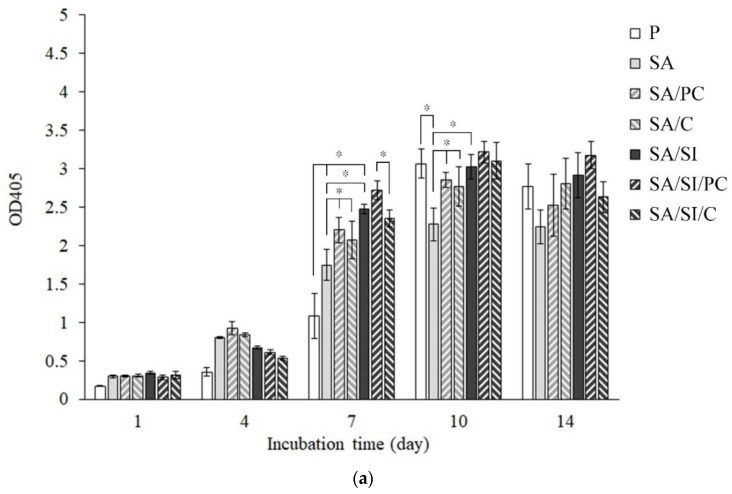

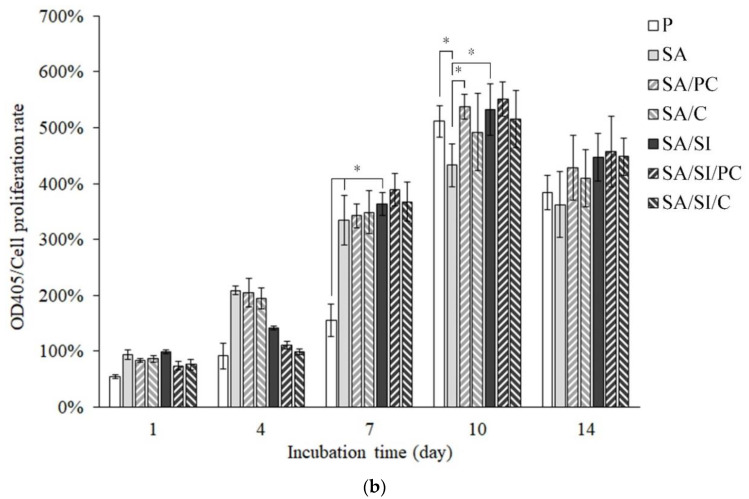
Progenitor bone cells of D1 were cultured on surface-modified PEEK for 1, 4, 7, 10, and 14 days: (**a**) total amount changes in ALP activity and (**b**) adjusted ALP activity in the unit cell (*n* = 6). Character symbols * indicate that each testing group after one-way ANOVA is significantly different (*p* < 0.05).

With the increase of cell culture time, the proliferation rate of cells had an upward trend and reached a peak on the seventh day (Figure 9). As shown in Figure 10, the proliferation rate no longer increased on the 10th day, and the secretion of ALP of cells was presumed to be increased, indicating that cell mineralization had begun; thus, such cells did not continue to proliferate. After 1 and 4 days of culture, the cell proliferation rate of the SA/SI/PC and SA/SI/C groups was higher than that of the other groups. The grafting of COL I could increase the cell proliferation rate primarily because of collagen hydrolysis, and the amino acids in COL I such as proline could promote cell proliferation [41,42]. Considering that the grafted phosphatidylcholine increased the hydrophilicity of the PEEK surface and hydrolysis into different fatty acids, the cells could easily attach to the surfaces [43]. After 7 days of culture, the proliferation rate of D1 cells on the SA group was the lowest because of the hydrophobic surface of SA. Accordingly, phosphatidylcholine had a better effect on promoting the proliferation of D1 cells than COL I.

#### 3.2.4. ALP Activity and Staining for the Mineralization of D1 Cells

ALP is a phospholipase secreted by osteoblasts, which can catalyze the hydrolysis of phosphomonoester R-O-PO_3_ to release phosphate and participate in the mineralization of bone matrix, while osteoblasts begin to differentiate, ALP will increase significantly. Therefore, determining whether osteoblasts are differentiated is possible by testing the content of ALP [44]. The ALP activity of D1 cells cultured on different surfaces of modified PEEK is shown in Figure 10. Use the reagent as the blank group as the reference (0%) for the determination. The higher the cell content, the higher the relative percentage. When the culture space is limited, the effect of cell proliferation will be inhibited. After the D1 cells were cultured for 14 days, although the surface of the PEEK sample had a 3D structure, there was a limitation of cell culture space. Since the osteoblast progenitor D1 cells were cultured in the study, D1 cells began to differentiate in large numbers and express ALP after the 7th day, and the cell proliferation process will not occur at this time. Therefore, the cell proliferation rate will not increase significantly. Although ALP was detected in 1 and 4 days of cell cultures, the activity of ALP did not increase significantly. After 7 days of culture, apart from the unmodified P group, the production of ALP in other modified groups had increased significantly [45,46]. The surface modification with grafting of active biomolecules remarkably promoted ALP secretion by D1 cells [47,48,49]. The amount of ALP produced after 10 days of cell culture reached a peak, indicating that the cells of all groups were undergoing early differentiation. Only the sulfonated group of SA had the lowest ALP secretion primarily because of the hydrophobicity of the sulfonated PEEK. By the 14th day of cell cultures, the cells had completed their differentiation; thus, the ALP content began to decrease.

**Figure 11:**
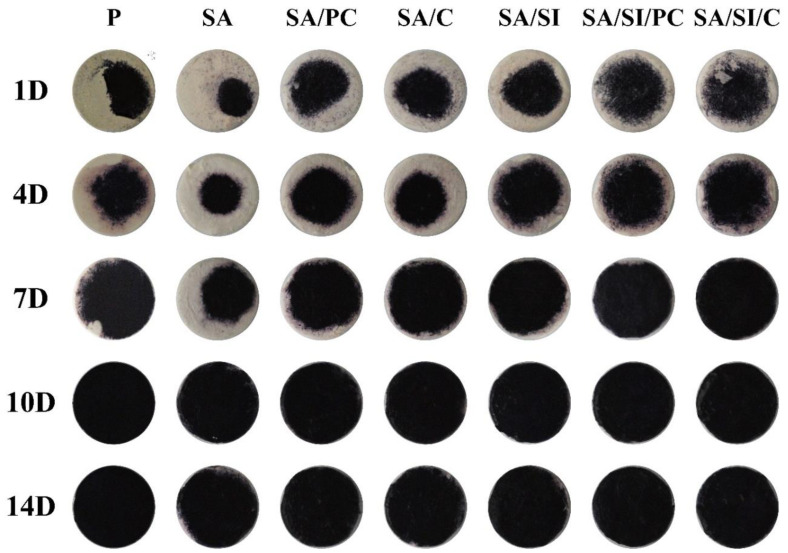
ALP staining results of osteo-progenitor D1 cells, which have been stained after culturing on surface-modified PEEK for 1, 4, 7, 10, and 14 days.

In addition to semi-quantitative testing, ALP could also be stained for qualitative analysis. The darker the color of the test piece, the more ALP was secreted by the cells. The ALP staining colors were shown in the concentrated area after 1 day of cultivation (Figure 11). This result indicated hydrophilicity, where cells cultured on the hydrophobic surface of SA had only a small distribution area, but PEEK grafted active biomolecules (SA/PC, SA/C, SA/SI/PC, and SA/SIi/C), and silylation (SA/SI) revealed widely distributed staining area. After 7 days of cell culture, except for SA, the cells of the other groups were almost covered with PEEK surfaces, and after 10 days of culture, the color was dark blue–purple, indicating that ALP activity reached the highest level on the 10th day (Figure 9).

### 3.3. Observations in In Vivo Testing

**Figure 12:**
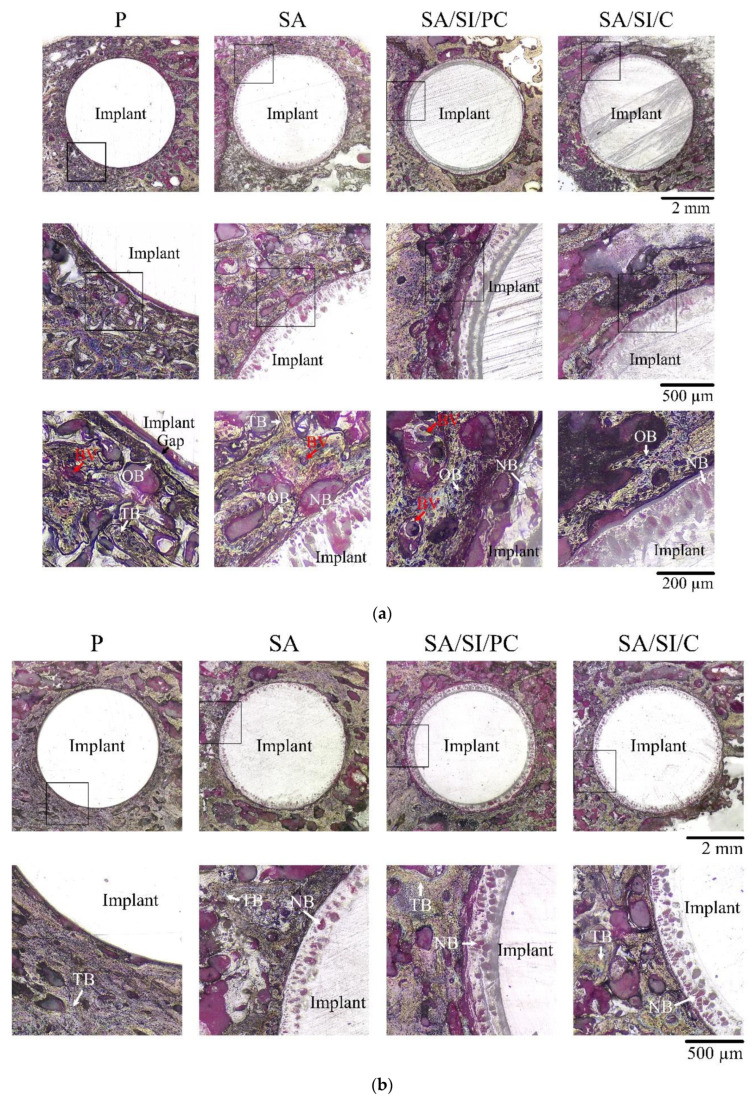

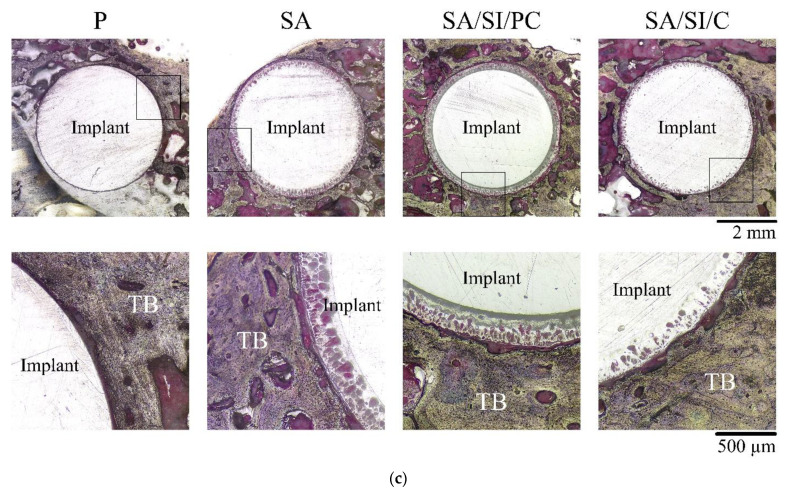
Optimal images of tissue after surface-modified PEEK was implanted into the rabbit distal medial malleolus: (**a**) 4 weeks after implantation, (**b**) 8 weeks after implantation, and (**c**) 12 weeks after implantation (BV: blood vessel, OB: old bone, NB: new bone, and TB: trabecular bone).

In the non-decalcified H&E-stained sections, after 4 weeks of implantation (Figure 12a), many osteoblasts and new blood vessels were found around the PEEK implant. For implants of unmodified bio-inert PEEK (P), gaps could still be observed between the tissues and edges of PEEK surroundings. However, for implants that had undergone surface modification of the SA, SA/SI/PC, and SA/SI/C groups, no gaps could be found between the tissues and implant groups. New bone could be seen integrating and growing into the modified surface of the implant. This in vivo result also showed that sulfonation caused the PEEK and surrounding tissue to exhibit better bond than the control P group. In the quantitative comparison by histology, the area of the new bone on the surface of SA/SI/PC implants was approximately 1.25 times higher than that of SA/SI/C, which was consistent with in vitro cell proliferation and ALP activity.

After 8 weeks of implantation (Figure 12b), the new bone around the implant had gradually matured into trabecular bone, and no gaps were observed even between P and bone tissues. The modified SA/SI/PC and SA/SI/C groups showed that the area of the new bone formed from sulfonated micropores increased with the increase of implantation time, but no significant difference was found between the two groups at 8 weeks of implantation. After 12 weeks of implantation (Figure 12c), the trabecular bone had been calcified, and all groups showed good osseointegration.

## 4. Conclusions

This study demonstrated the use of sulfonation, silylation, and grafting of different active biomolecules to improve the surface and biological performances of PEEK. After sulfonation, the surface of PEEK fabricated a certain thickness with an inter-connective microporous structure. After silylation of the sulfonated PEEK, the amount of surface grafting active biomolecules increased, indicating that COL I and phosphatidylcholine could be effectively grafted, and the accumulated weight of phosphatidylcholine was relatively large. In vitro, the cell viability showed that PEEK after different surface modifications were all not toxic to L929 cells. The PEEK surfaces of active biomolecules grafted after silylation promoted the adhesion and proliferation of D1 cells and secretion of ALP because of the enhancement of roughness and hydrophilicity. In vivo, PEEK after sulfonation improved bone formation, showing a 3D inter-connective porous structure. Comparing the two active biomolecules grafted (COL I and phosphatidylcholine), the results of cell proliferation in vitro and tissue sectioning in vivo indicated that phosphatidylcholine had a better effect on promoting cell proliferation and differentiation, thereby allowing better osseointegration with the host bone tissues.

## Data Availability

The data presented in this study are available on request from the corresponding author.
